# Parents in science

**DOI:** 10.1186/s13059-018-1549-3

**Published:** 2018-10-29

**Authors:** Emily Perry, Kristin Tessmar-Raible, Florian Raible

**Affiliations:** 1 European Molecular Biology Laboratory, European Bioinformatics Institute, Wellcome Genome Campus, Hinxton, Cambridge, CB10 1SD UK; 2 Max F. Perutz Laboratories, University of Vienna, Vienna, Austria

## Abstract

As part of our Q&A series, *Genome Biology* spoke to four scientists about their personal experiences as parents in their careers to highlight the challenges of researchers having children and the support they need in this regard. Our participants also included a couple (Kristin Tessmar-Raible and Florian Raible), as we were interested to know whether both parents being active researchers can have an impact. One of our participants wishes to remain anonymous.

**Please give us a very brief overview of your research interest.**

**Emily Perry (EP)**: I work as an outreach project leader for Ensembl at EMBL’s European Bioinformatics Institute (EMBL-EBI). I run a small team that delivers training on using the Ensembl genome browser and associated tools, as well as answering help emails, producing online help and documentation, and running UX on the site and social media. I came into this after a PhD looking at chromatin compaction in Cornelia de Lange syndrome.

**Anonymous participant (AP)**: I am a bioinformatics scientist who works with wet-lab scientists to develop new protocols and technologies for sequencing applications.

**Kristin Tessmar-Raible (KTR)**: In general, I am interested in understanding how light and endogenous timers (also called clocks) impact on the physiology and behaviour of animals, and how these processes interplay with the natural ecology of animals. More specifically, I am especially focussing on the moon and the endogenous clocks that are entrained by it.

**Florian Raible (FR)**: I study the hormone systems involved in growth, reproduction and regeneration of animals, and their effect on stem and germ cells. Much of our work focuses on a marine worm in which hormones literally decide between life and death: depending on the hormone status, these animals can either grow and regenerate, or they will prepare for reproduction, which is directly followed by death.

**At what stage of your career did you have children? Was your career a factor in deciding when to have them?**

**EP**: My daughter is only one and a half years old, so I had her while in my current job. I was previously a team member in my current team, and my job involved significant amounts of travel, going out to research institutes around the world to deliver training. I knew that having a child and living that kind of lifestyle would not be compatible, so I held off for a couple of years to get into a management position. Now instead of delivering as much training, I focus more on arranging for other people to deliver it, and work on piloting new projects at home. When I was promoted to management, we were somewhat short-staffed, so I chose to wait until I had recruited and trained up my new team members before trying to get pregnant.

**AP**: My first child was 5 months old when I began my MSc and 9 years when I finished my PhD. My second child was not that carefully planned, so career was not a factor in either.

**KTR**: Till almost the end of my postdoc, I really didn’t want to start a family. It was always science first. But there was a moment during the postdoc time when I started to wonder if science is really everything in life, especially given that both my spouse and myself come from relatively large families. Being from eastern Germany helped me to have the confidence that combining a career and a family will be possible [1].

**FR**: We decided to have children when we were both at the transition between the postdoc and the group leader stage. Our first child was basically born in the midst of a series of job interviews. We both did not want to compromise our personal productivity during the PhD and postdoc phase, and we probably also didn’t yet feel ready to have children.

**How did being a parent impact your career?**

**EP:** Most of the change has been in myself. I prioritize differently, and am more strict in saying that I won’t stay late or work weekends if there is an alternative. I sometimes have conference calls with colleagues in California, and instead of staying an hour later at work (which would mean sorting out childcare arrangements for picking up my daughter), I’m more likely to arrange them later in the evening after she goes to bed.

I’ve found everybody to be very accommodating and don’t think I’ve experienced any discrimination. I think a significant factor is that my boss is a dad himself, and his wife has a career as well as their two sons, so I think he understands.

**AP**: Before I finished my PhD, my son began to show the early symptoms of childhood depression. I had already decided not to do a postdoc. I did not love academia, but I also had to begin saving for my son’s college and doing a postdoc that paid $30 K less than a scientist job would have put my education ahead of his. So, I took a staff scientist job at a research institute.

Over the next couple of years, my son’s illness became progressively worse until he became very depressed and often suicidal. I honestly didn’t know if he would survive it. During this time, I was not able to work long hours and a lot of my mental energy was drained by things that were happening outside work. A few people at work knew what was happening and they were very supportive. When my son’s illness was at its worse, I was working with a wet-lab colleague who was a very positive person and we were doing interesting projects. My career was a distant second priority in my life, but being able to focus on something else made the first priority easier to cope with.

Then, one day, my son got better. I don’t really know why. He fought really hard but he had been fighting for years. It could have been a good medication kicking in or a bad one wearing off. He said it was like turning off a faucet. Being released from that enabled me to focus more on my career, and when my colleague left the institute I moved into industry. This was partly for a change, but also partly because by then I also had huge day-care expenses (which completely wiped out the salary boost from being a staff scientist) and a second college education to save for. Kids are great but they’re very expensive.

**KTR**: As a scientist, my response is that, strictly speaking, I can’t really tell, because I am lacking the control sample (i.e., a clone of myself without children). In practice, where I do feel an impact is that having kids “smoothens” a bit the sometimes pretty steep ups and downs that the everyday scientific life throws one into. It hopefully also makes me a better mentor.

**FR**: Adding to the notion of “smoothening” extremes, being a parent inevitably requires stricter time keeping. School opening hours and holidays simply limit the enormous freedom we enjoyed as students and postdocs to work at unconventional times. It may have helped that our first child coincided with the transition to a group leader position, where personal experimental work became less of a defining criterion for success, in favor of more “portable” tasks. I still am a late worker, but spending significant time on the computer is no big deal after the children are asleep.

**Have you experienced any bias towards colleagues who don’t have children?**

**KTR**: No, I haven’t experienced any bias, either positive or negative.

**FR**: Not in the sense of a personal bias, but I think for example that in the context of interviewing faculty candidates, we are much more sensitized to the issues that come with parenting. For a person with children, for instance, the question of if or not a position comes with a tenure-track option makes much more of a difference than for a person who carries less personal responsibility. If an institution is blind to this, they risk losing excellent candidates, especially female ones.

**Did you experience any restriction regarding attending conferences?**

**EP**: I’ve only had a few trips away since my daughter was born and I’ve hated being away from her—I suspect it will get easier as she gets older. The first time I went away I was still breastfeeding so I was having to nip out and pump milk at various times during the trip, which was okay because I had a hotel room right beside the meeting room.

Since I’m usually delivering talks or training when I go away, there’s no option to bring my daughter with me. I know that some conferences offer childcare, but none of the ones I’ve been to since she’s been around. Even if I did bring her, taking care of a jet-lagged child by myself, whilst staying in a hotel and having meetings and talks at unusual times is not something I’d want to do. At least now there is Skype and I can see her every day.

**AP**: No. My husband takes care of our kids when I go away. We need to listen to the voices of the people who do not have that option.

**KTR**: The single exception I recall was not being allowed on a flight to a conference in Japan during late-stage pregnancy. Fortunately, in this instance, the topic of the conference was such that Florian was able to substitute for me.

**FR**: We mutually share our electronic calendars. Fortunately, we have so far managed pretty well to attend any conference or meeting that either of us was seriously interested in. That said, we sometimes need to be pragmatic and cut down attendance at longer conferences to a more narrow time slice. The other very important component here is a supportive environment. The campus kindergarten is at breastfeeding distance, and both school and kindergarten offer afternoon support. Moreover, even though the grandparents live more than 600 km away, they have been able to help out at times when we had simultaneous obligations. Fortunately, as dual career scientists, we both have pretty flexible working hours or can occasionally work from home.

**Has there been any improvement with this respect?**

**KTR**:

Yes, there have been significant improvements to help supporting scientists with kids. When the topic of “parenting scientists” started to reach a certain awareness level in the scientific community, conferences started to offer on-site support for children. While this is an encouraging move, there are still many situations that are difficult to handle. For example, older children who are already in school cannot join their traveling parents, while younger kids may not really accept being supervised by unfamiliar persons.

A promising development that may help to ease these issues is the idea to offer monetary support that can be used in a more flexible way, be it for paying for more hours to a nanny back at home, or a flight ticket for one of the grandparents to accompany the parenting scientist to the conference. The European Molecular Biology Organization (EMBO) has started to offer this type of flexible conference support to its EMBO-YIP fellows and former fellows. From our own experience we can say that this is truly helpful.

**Is there anything else that can be improved to help the situation?**

**FR**: Following up on this argument, it would certainly be helpful if other organizations considered such flexible schemes as well, making them more accessible to a wider range of parenting scientists.

**What kind of support do you wish you could have had access to?**

**EP:** I’ve found everybody so supportive; I think I’m pretty lucky in that regard. When I first went back to work I was still breastfeeding, and I was able to pump at work due to a space being put aside for it, including a locking door, a comfy chair and fridge for your milk. We also have a nursery on campus, but it’s massively oversubscribed so we didn’t get a space (even though I contacted them within a couple of weeks of finding out I was pregnant). At first I was disappointed, but now I’m glad my daughter is somewhere nearer home, where both my husband and I can easily pick her up and drop her off, and she has friends who live locally.

**AP**: The people in my workplace actually provided the best source of support that I had. My friend, who has a son with autism, formed a support group for employees who were parents of children with special needs and told me to go. Until then, almost everyone in my life told me what I was doing wrong. A lot of my son’s behavior looked like insolence and people kept telling me that it could be solved with better parenting. It couldn’t and the advice was very hurtful. The parents in the group did not do that. There was a lot of listening and, if there was any advice, it was practical and useful, like how to fight with schools for services. It was what you would expect from compassionate parents who were also trained to be practical problem solvers and to know what they did not know. It kept me from cracking up and allowed me to be the mother that my son needed. I had access to this type of support because our institute was committed to their employees, and it did help me to be more productive at work. My wish would be for all parents who need this kind of support to have access to it.

**KTR**: Ideally, the time-turner gadget from the third Harry Potter book ☺. More seriously, I think all full-time working parents, no matter which profession they have, will gladly take as much household and high-quality (!) nanny support as they can get. There is never enough of this.

**Was there anything you wished to do in your career but couldn’t due to having children?**

**EP**: Not so far, but I’ve only been doing this a short while.

**AP**: I was less productive when my son was depressed, obviously. Because his illness was at its worst a few years after I finished my PhD, if I had gone into academia I never could have done the work it would have taken to become tenured. I don’t have loads of publications and maybe I would be further along in my career now if he hadn’t been sick for those years. So what? My son is alive. No one took away my credentials. I still get to do science and someday maybe something I do will give another parent the experience of having a child who suddenly gets better. A career outside of academia doesn’t have to be a sprint. It can be a marathon and there is no finish line anyway.

**KTR**: At the moment I do sometimes miss the long working days where I could mull over a scientifically puzzling question till midnight or spend the entire weekend working on a paper not caring about the time. But with the kids growing up, I anticipate that these times will gradually come back again. And of course, I had to give up other non-scientific hobbies, like martial arts or horseback riding. But looking at our kids, I feel that these are the right priorities, because being a parent has a lot of emotional rewards and gives inner strengths.

**FR**: I also believe this is a bit like a trade: children mean giving up individual freedom (which, besides personal hobbies, would include scientific field trips, or research sabbaticals that would be difficult to organize in this setting). But you also surround yourself with little scientific role models: children are true experts in something that we would call curiosity-driven research.

**Do you think there is any difference between men and women as parents and the impact on their career?**

**EP:** I think that the expectation on men and women is different. It’s expected that women’s lives change when they have children, that they won’t stay at work as late as they did before, that they won’t travel as much as before or they won’t come in at weekends. However, men are expected to just carry on as before. Certainly with my husband, I find that he gets asked to stay late or travel at inconvenient times, and it never occurs to anyone that this may be unreasonable because he’s a parent, whereas everybody I work with seems to accept that I’m not going to be doing that (sample size of two, so I don’t think we can draw any real conclusions here though). The positive for me, at the moment, is that I’m able to dictate how and when I work in a way that doesn’t have a negative impact on my parenting. The danger, however, is whether this will make a long-term impact and my relative unavailability will hold me back in future.

**AP**: In my experience, the differences vary a lot by where you work and the individuals you work with. But I am jealous of how men can parent openly without having to worry that it will become a “thing”. For example, I had a male colleague who had a cheap but unreliable nanny. He was always leaving to watch his kids and he never apologized for it. He just left. I would love the freedom to do that, but it seems to be a male prerogative. Women have to worry more that they will be seen as uncommitted. Also, men are more likely to have a stay-at-home spouse and never have to leave for their kid’s dentist appointment. For most women this is an unimaginable luxury.

**KTR**: Well, men and women have brains that are differently wired, different hormones at different levels; thus, I think it is very likely that men and women will behave differently as parents. But diversity does not imply that one is doing it better than the other.

Concerning the impact of parenting on careers, based on my experience this is a complex topic. My impression is that for women some switch to a “family-is-all-and-science-is-not-important-any-longer” phenotype, while others try to balance between family and science. For those who try to balance, having kids is not necessarily a career disadvantage. This is especially due to the fact that committees are much more aware of the topic (and of female applicants in general). Also, there are official time extensions given for the eligibility to high-profile European grants and programs, such as ERC-StG or the EMBO-YIP program. I don’t know, however, if this is equally true outside Europe. For fathers that take over an equal share of the family workload, the situation is unfortunately rather different. Except when they officially take time off for parenting, I feel that they do not get equal acknowledgement to mothers. More fairness here would be important. Why not extend the eligibility deadlines for fathers, too, if for instance the spouse confirms that the work-load is shared equally?

**Is it helpful when both parents are researchers? Does it make it harder or easier?**

**EP:** I think having a degree of understanding between both parents about the pressures of your respective careers is important. You don’t have to be in the same field to understand that your partner may have work commitments that impact on your parenting. My husband is a software engineer, but we both understand how important our careers are to us, which means that we make sacrifices for one another and our daughter when we need to.

If you’re working in the same field, I can see how it would be very difficult when important events that you both have to attend happen, because you have to sort out some kind of childcare. This is particularly difficult as scientists, since we move about and most of us don’t have convenient relatives, or even friends we’ve known for more than 5 years, living nearby. However, going back to my point before about attending a conference alone with a child and making use of conference childcare, I would be more likely to do that if my husband was attending the same conference as I was.

**AP**: It was helpful to me that my husband was *not* a researcher. My son’s day-care expenses were more than half of my PhD stipend. I was only able to go to graduate school because my husband had a job that paid enough to support our family, which would not usually be possible until researchers are many years into their career. I think a lot about the lost potential of people who cannot enter the field or go as far as they are capable of going because they do not have the money that it takes to do the training. A lot of those people are parents.

**FR**: I believe that on a personal level, compared to a setting with more different professions, we are probably more likely to support each other when the profession requires extra time-off hours. I mean, if I did not know how relevant a paper revision or a grant deadline really can be, I might not be as inclined to take over the kids for a full weekend, and vice versa. In our specific case, as our research is close enough for us to understand each other’s work, we have the additional benefit of being able to discuss science with each other whenever there is time. I am curious when the kids will start contributing to these discussions…

**What was your biggest challenge in your career as a parent?**

**EP:** Too early to say, but the thing I worry about most is job transitions. My contract is limited, and I know any job that I’m likely to go into in future may also have a fixed-term contract. I’m trying to build up my savings so that if I do have a short time out of work, I’ve got a buffer zone. I also don’t want my daughter to have the kind of childhood where she gets uprooted and moves schools every few years. I had to move schools once as a child and it was really tough. I’m hoping that being where we are (Cambridge) I’ll be able to stay put when I move into my next job.

**AP:** After I left the institute, I worked for an understaffed biotech where the hours were miserable, for example requests on Saturday night for huge analyses due Sunday night. For a year, I was in a continuous state of panic about being able to get everything done and it interfered with my ability to be a good mother. When I complained, my boss told me I had a bad attitude. So I quit.

If you have marketable skills, like bioinformatics, you can do this. When I talk to young women scientists, I try to convince them that it is as easy to fall in love with a rich field as a poor one, and it makes the rest of your life so much easier. If you are exhausted, you can order take out. If your kid needs a tutor, you hire one. I don’t want them to ever be afraid to enter a field that is majority male if it is the right fit for them. I even stole two of my wet-lab colleague’s undergraduate women students and convinced them to become dry lab. Though I may have gone too far. One has joined a hedge fund.

**KTR**: An important job interview involving a transatlantic flight that we almost needed to cancel, as our first daughter caught an aggressive virus causing stomach sickness shortly before the trip. When she recovered in time, it all seemed fine, but being still rather unexperienced parents, we didn’t know yet that these viruses typically hit the parents as well, with a few days of delay, which meant right at the trip. Professionally, the trip was a success, resulting in a dual job offer, but it was a really special challenge.
